# The complete chloroplast genome sequence of *Libanotis buchtormensis* (Apiaceae)

**DOI:** 10.1080/23802359.2021.1997120

**Published:** 2021-12-28

**Authors:** Ping Wang, Wei-dong Fan, Yi-Jie Qiu, Nan Geng, Yi-hua Dong, Li Huai-Zhu, Wen-bo Wang

**Affiliations:** a Department of Life Sciences, Xianyang Normal University, Xianyang, China; b Qinling National Botanical Garden, Xi’an, China

**Keywords:** Chloroplast genome, *Libanotis buchtormensis*, phylogeny

## Abstract

*Libanotis buchtormensis* (Fisch.) DC. is one of the traditional Chinese herbal medicines in the Qinling District, while the genetic information is limited. In the study, we reported the complete chloroplast genome of *L. buchtormensis* using BGISEQ-500 sequencing data. The complete chloroplast genome was 147,036 bp in length with a GC content of 37.6%, consisting of four parts, namely LSC (91,969 bp), SSC (17,469 bp), and two IRs (18,799 bp in each). The cp genome contained 127 genes, including 83 CDS genes, 36 tRNA genes, and 8 rRNA genes. The phylogenetic analysis showed that *L. buchtormensis* was sister to *Ledebouriella seseloides*.

*Libanotis buchtormensis* (Fischer) Candolle 1829, a perennial and subshrubby herb belonging to the Apiaceae family, is one of the Taibai herbal medicines in the Qinling district and is commonly known as ‘chang chun qi’ (Wang et al. 2015). Traditionally, the root of the species is generally employed to treat rheumatism, articular pain, and the cold (Wu and Zhang 2009). Pharmacological studies indicated that osthole, the primary bioactive compound of *L. buchtormensis*, is a promising natural composition exhibiting immunomodulation, antioxidant, anticancer and antimicrobial activities (Zhang et al. 2015). In the wild, *L. buchtormensis* prefers the sunny rocky slopes at altitudes of 700–3000 m with scattered natural populations (Wang et al. 2015). Since the genetic information of *Libanotis* is limited, the complete chloroplast genome of *L. buchtormensis* is helpful to clarify its phylogenetic position and enlarge the further evolutionary studies. Furthermore, the result will provide theoretical basis for the protection of the species.

Total genomic DNA was extracted from the fresh leaves of *L. buchtormensis* with the modified CTAB method (Doyle and Doyle 1987). The sampling was collected from Zhouzhi County, Shaanxi Province, China (N33°48′37.76″, E108°23′4.92″). The DNA sample (No. YF001) and the voucher specimen (No. YF20200705) were deposited at Xianyang Normal University (http://www.xysfxy.cn/, Huaizhu Li, lihuaizhu121@126.com). The extracted genomic was fragmented to an average size of 200–400 bp for sequencing library preparation. The paired-end sequencing was performed on the BGISEQ-500 platform. Clean data obtained using SOAPnuke software (Chen et al. 2017) was assembled by GetOrganelle pipeline (Jin et al. 2020) with that of *Seseli montanum* (Genbank KM035851) as a reference sequence and then annotated by Geneious Prime (Kearse et al. 2012). The total chloroplast genome of *L. buchtormensis* was submitted to Genbank with accession number MZ707534.

The complete chloroplast genome of *L. buchtormensis* displayed a standard angiosperm structure of 147,036 bp in length, comprising a large single-copy region (LSC, 91,969 bp), a small single-copy region (SSC, 17,469 bp), and a pair of inverted repeat regions (IRs, 18,799 bp in each). The chloroplast genome harbored a total of 127 genes, including 83 protein-coding genes (CDS), 36 transfer RNA (tRNA) genes, and 8 ribosomal RNA (rRNA) genes. The GC content of the plastid genome was 37.6%.

To investigate the phylogenetic position of *L. buchtormensis*, a phylogenetic tree was constructed together with 31 other complete chloroplast genome sequences of Apiaceae species. All sequences were aligned by MAFFT (Katoh and Standley 2013) and checked manually in Geneious Prime (Kearse et al. 2012). Then, a maximum-likelihood (ML) analysis (with 1000 bootstrap replicates) was performed using MEGA-X (Kumar et al. 2018). The results exhibited that *L. buchtormensis* was clustered with *Ledebouriella seseloide*s with high support (Figure 1). Meanwhile, the species showed a close relationship to *Peucedanum japonicum* and *Seseli montanum* (Figure 1). The complete cp genome of *L. buchtormensis* will offer a genetic background for research and conservation of *L. buchtormensis*.

**Figure 1.**
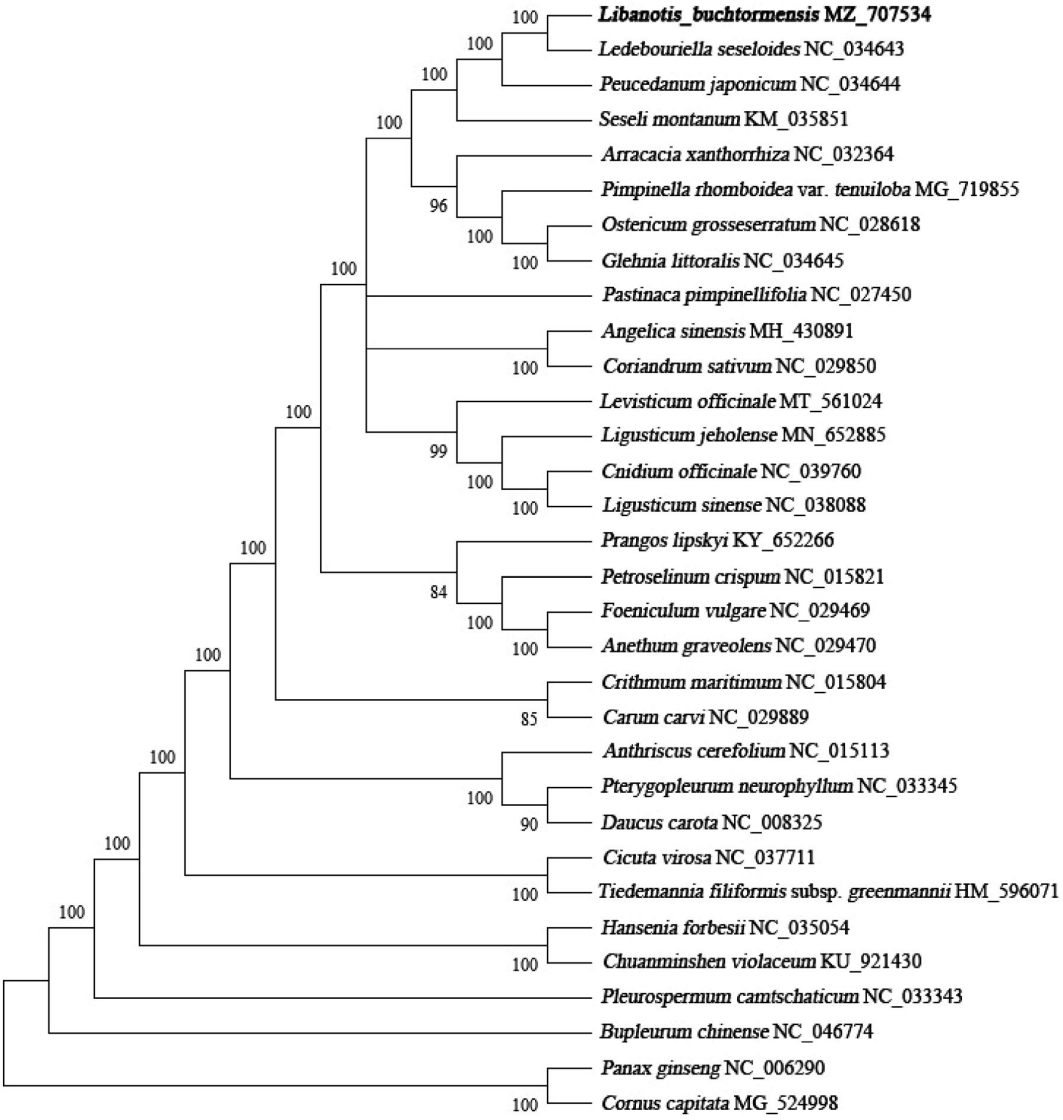
Phylogenetic position of *L. buchtormensis* inferred from maximum likelihood (ML) method based on 32 chloroplast genome sequences. The bootstrap support values were shown on the branches.

## Data Availability

The genome sequence data that support the findings of this study are openly available in GenBank of NCBI at https://www.ncbi.nlm.nih.gov under accession No. MZ707534. The associated BioProject, SRA, and BioSample numbers are PRJNA752500, SRR15357742, and SAMN 20599686, respectively.
